# A Practical Handbook of Medical Chemistry

**Published:** 1850-12

**Authors:** 


Art. IX.—*A Practical Handbook of Medical Chemistry.* By John E. Bowman, Demonstrator of Chemistry in King's College, London; and author of "Practical Chemistry." Philadelphia: Lea & Blanchard. 1850.

Mr. Bowman says: "the want which, as a teacher of Practical Chemistry in a medical school, I have long felt, of a small manual containing instructions for the examination and analysis of urine, blood, and a few other of the more important animal products, both healthy and morbid, and comprising also directions for the detection of poisons in organic mixtures and in the tissues, was my chief inducement in undertaking to write the present little work.

"In doing this, my endeavor has been to supply a book that will be found useful, not only to the medical student, but also to the practitioner, to whom the value and importance of the applications of modern chemistry and microscopic analysis to his art are becoming daily more and more apparent."

Every one who knows anything of the value of the means of information referred to in Mr. Bowman's remarks, must be gratified to know that a book so imperatively demanded by the wants of the profession, has at length been published. The time is coming when the medical practitioner will be obliged either to have the practical knowledge, taught in this book, under ready command, or take his place at the foot of the profession. One of the good effects of the violent pressure of competition, that is felt now among medical men in the practice of their profession, is the necessity it will impose for attaining the highest possible amount of qualification for grappling with disease. Pretension, ignorance, and impudence always carry within them the seeds of consumption—they are born with tubercles, beyond the reach of even authors who cure consumption. The thoroughly qualified physician, one armed at all points with information which can be readily used for every emergency, will always bear off the palm from the mountebank, and the charlatan. Chemical analysis, and the use of the microscope are getting to be as essential to the practitioner of medicine, in the investigation of dis-eased action, as any other means of diagnosis, and he that fails to make himself familiar with these matters must lose caste in his profession, and be distanced by those who know how to improve themselves up to the requirements of their profession. An attempt, an abortive one of course, has been made latterly to scowl and bluster down all efforts for educating the mind for the profession of medicine, and a broad invitation was thrown out for clowns, of every shade of ignorance to rush to the temples of Esculapius, and adorn its shrine with their presence, provided votive offerings were laid upon the altars. Against such absurdity, nay treason itself to the profession, all intelligent minds should enter their protest. The doctrine is at war with every interest of society; it is calculated to ruin the dupes who are shallow enough to mistake the braying for music; it is destructive to the progress of mind, degrading to the medical profession, and a serious injury to the public who may be the prey of men who are so defective in education as to be unable to read either a medical book, or to understand a lecture that has any important information in it.

We hail the appearance of Mr. Bowman's work with a sincere satisfaction. It cannot fail to do good, provided it is studied with diligence, and practiced with ability. We present the reader the principal contents of the book:

Part I.—Healthy urine—quantitative analysis of healthy urine, average composition of healthy urine; morbid urine; qualitative examination of urine suspected to contain either an unnatural proportion of some one or more of the usual ingredients, or some abnormal matter. Examination of urine, the nature of which is altogether unknown. Quantitative analysis of Diabetic urine.—Quantitative analysis of albuminous urine.

Part II.—Urinary Calculi. Qualitative examination of urinary calculi, the composition of which is unknown. Biliary calculi, or gall stones. Gouty concretions.

Part III.—Blood. Healthy blood. Quantitative analysis of blood. Morbid blood.

Part IV.—Milk, mucus, pus, bone, etc. Quantitative analysis of milk. Milk during disease. The adulterations of milk. Mucus. Pus. Bone. Examination of mixed animal fluids.

Part V.—The detection of Poisons in organic mixtures, etc. Arsenic, Antimony, Mercury, Lead, Copper. Detection of zinc in organic mixtures and in the tissues. Iodine, Sulphuric Acid, Hydrochloric Acid. Quantitative determination of Hydrochloric acid. Nitric acid. Oxalic acid. Hydrocyanic acid. Opium. Method of examining an organic mixture suspected to contain some mineral poison, the nature of which is unknown.

The medical practitioner cannot fail to perceive, in this range of investigation, how very important an agent such a book as Mr. Bowman's must be. We commend it to their attention, careful study, and diligent practice.

